# Description of *Polystyrenella longa* gen. nov., sp. nov., isolated from polystyrene particles incubated in the Baltic Sea

**DOI:** 10.1007/s10482-020-01406-5

**Published:** 2020-04-01

**Authors:** Stijn H. Peeters, Sandra Wiegand, Nicolai Kallscheuer, Mareike Jogler, Anja Heuer, Mike S. M. Jetten, Christian Boedeker, Manfred Rohde, Christian Jogler

**Affiliations:** 1 Department of Microbiology, Radboud Universiteit, Nijmegen, The Netherlands; 2 Institute for Biological Interfaces 5, Karlsruhe Institute of Technology, Eggenstein-Leopoldshafen, Germany; 3 Department of Microbial Interactions, Institute of Microbiology, Friedrich Schiller University, Jena, Germany; 4 Leibniz Institute DSMZ, Brunswick, Germany; 5 Central Facility for Microscopy, Helmholtz Centre for Infection Research, HZI, Brunswick, Germany

**Keywords:** Marine bacteria, *Planctomycetes*, *Planctomycetaceae*, Marine biofilms, Microplastic particles

## Abstract

Planctomycetes occur in almost all aquatic ecosystems on earth. They have a remarkable cell biology, and members of the orders *Planctomycetales* and *Pirellulales* feature cell division by polar budding, perform a lifestyle switch from sessile to motile cells and have an enlarged periplasmic space. Here, we characterise a novel planctomycetal strain, Pla110^T^, isolated from the surface of polystyrene particles incubated in the Baltic Sea. After phylogenetic analysis, the strain could be placed in the family *Planctomycetaceae*. Strain Pla110^T^ performs cell division by budding, has crateriform structures and grows in aggregates or rosettes. The strain is a chemoheterotroph, grows under mesophilic and neutrophilic conditions, and exhibited a doubling time of 21 h. Based on our phylogenetic and morphological characterisation, strain Pla110^T^ (DSM 103387^T^ = LMG 29693^T^) is concluded to represent a novel species belonging to a novel genus, for which we propose the name *Polystyrenella longa* gen. nov., sp. nov.

## Introduction

Planctomycetes are bacteria with a Gram-negative cell envelope architecture belonging to the PVC superphylum, along with *Verrucomicrobia*, *Lentisphaerae*, *Kiritimatiellaeota*, *Candidatus* Omnitrophica and *Chlamydiae*. Many representatives of this superphylum have major medical and biotechnological relevance (Wagner and Horn 2006), and can play key roles in global biogeochemical cycles (Peeters and van Niftrik 2018; Strous et al. 1999; Wiegand et al. 2018).

Planctomycetes are found in many environments, e.g. on (marine) algae (Bengtsson and Øvreås 2010; Bengtsson et al. 2012; Bondoso et al. 2014, 2015, 2017; Lage and Bondoso 2014; Vollmers et al. 2017), in peat bogs in northern wetlands (Kulichevskaya et al. 2012) or in hot springs (Slobodkina et al. 2015). They are assumed to metabolise complex carbon substrates released by biotic surfaces (Frank et al. 2014; Jeske et al. 2013; Lachnit et al. 2013; Wiegand et al. 2018). In the past, Planctomycetes were thought to have a number of exceptional traits (Devos et al. 2013; Devos and Reynaud 2010; Fuerst and Sagulenko 2011; Fuerst and Webb 1991; König et al. 1984; Lindsay et al. 1997; Lonhienne et al. 2010) that would place them at the base of the eukaryotes, but these properties have been re-interpreted in the recent years (Acehan et al. 2013; Boedeker et al. 2017; Jeske et al. 2015; Jogler 2014; Jogler et al. 2011; Jogler and Jogler 2013; Neumann et al. 2014; Rast et al. 2017; Rivas-Marin et al. 2016; Santarella-Mellwig et al. 2013). The cell envelope architecture of Planctomycetes was classified as Gram-negative (Boedeker et al. 2017; Devos 2014; van Teeseling et al. 2015).

Nevertheless, Planctomycetes are non-canonical as they have many remarkable properties. They perform cell division by budding or binary fission (or even a combination) while lacking most of the canonical cell division proteins, including FtsZ (Jogler et al. 2012; Pilhofer et al. 2008; Wiegand et al. 2020). Many Planctomycetes can perform a lifestyle switch between sessile mother cells and motile daughter cells (Jogler et al. 2011; Wiegand et al. 2020). They can also have unusual crateriform structures on their cell surfaces, which are visible by electron microscopy. Their periplasm is enlarged and forms large invaginations into the cytoplasm. Several planctomycetal strains are regarded as potential talented producers of as yet uncharacterised small bioactive molecules (Graça et al. 2016; Jeske et al. 2016; Wiegand et al. 2018). Many Planctomycetes possess large numbers of giant genes (Kohn et al. 2016; Reva et al. 2008) and are amongst the bacterial phyla with the highest number of predicted genes of unknown function (Overmann et al. 2017; Wiegand et al. 2018).

In this study, we describe a novel strain, Pla110^T^, which was isolated from polystyrene particles incubated in the Baltic Sea. The strain is part of the family *Planctomycetaceae*, which includes the limnic model planctomycete *Planctopirus limnophila*. Most of the other known members of this clade are marine bacteria.

## Materials and methods

### Isolation and cultivation

Strain Pla110^T^ was collected during a sampling campaign, in which polystyrene particles were stored in small incubators, submerged in water and incubated at the given location for 14 days. The strain characterised here was isolated from the Baltic Sea 120 m off the shore at Heiligendamm, Germany (54.146 N 11.843 E). Isolation was performed as previously described (Wiegand et al. 2020).

The strain was cultivated and analysed in M1H medium supplemented with *N*-acetyl-glucosamine (NAG) and artificial seawater (ASW) (M1H NAG ASW medium) as described earlier (Wiegand et al. 2020) and incubated in a shaking incubator at 28 °C and 110 rpm. Medium supplemented with 1.5% (w/v) washed agar was used for plates.

### Light microscopy and electron microscopy

Phase contrast micrographs were taken with a Nikon Eclipse Ti inverted microscope and a Nikon DS-Ri2 camera. To ensure good image quality, specimens were immobilised using a 1% (w/v) agarose cushion and MatTek glass bottom dishes (35 mm, No. 1.5) (Boedeker et al. 2017). ImageJ (Rueden et al. 2017) was used to examine cell size by sequentially applying an Otsu threshold, a watershed, and count particles.

Field emission scanning electron microscopy was performed as described (Boersma et al. 2019). Briefly, bacteria were fixed in formaldehyde, washed, and placed on cover slips coated with poly-l-lysine solution. Cover slips were then fixed in 1% (v/v) glutaraldehyde and washed twice before dehydrating in a graded series of acetone (10, 30, 50, 70, 90, 100% (v/v)) on ice. Samples from the 100% acetone step were brought to room temperature before placing them in fresh 100% acetone. Samples were then subjected to critical-point drying with liquid CO_2_ (CPD 300, Leica). Dried samples were covered with a gold/palladium (80/20) film by sputter coating (SCD 500, Bal-Tec) before examination in a field emission scanning electron microscope (Zeiss Merlin) using the Everhart Thornley HESE2 detector and the inlens SE detector in a 25:75 ratio at an acceleration voltage of 5 kV.

### Physiological analyses

Determination of the pH optimum for growth was performed at 28 °C, with buffering agents 100 mM 2-(*N*-morpholino)ethanesulfonic acid (MES) at pH 5 and 6, 100 mM (4-(2-hydroxyethyl)-1-piperazineethanesulfonic acid) (HEPES) at pH 7, 7.5 and 8, or 100 mM *N*-cyclohexyl-2-aminoethanesulfonic acid (CHES) at pH 9 and 10. Temperature optimum for growth determination was performed at pH 7.5 with temperatures ranging from 10 to 40 °C. Cell densities were inferred from optical density measurements (OD_600_).

### Genome information

The genome and 16S rRNA gene sequence of strain Pla110^T^ are available from GenBank under accession numbers CP036281 and MK554533, respectively. Information on genome sequencing and assembly was previously published (Wiegand et al. 2020).

### Phylogenetic analysis

16S rRNA gene-based phylogeny was computed for strain Pla110^T^, the type strains of all described planctomycetal species (assessed in January 2020), all isolates recently described (Boersma et al. 2019; Kallscheuer et al. 2019a, b, c, d, 2020; Kohn et al. 2019; Peeters et al. 2019; Rensink et al. 2020; Wiegand et al. 2020) and with an outgroup of strains from outside the *Planctomycetes*, but part of the PVC superphylum. The alignment of 16S rRNA genes was made with SINA (Pruesse et al. 2012). Phylogenetic analysis was performed employing a maximum likelihood approach with 1,000 bootstraps, the nucleotide substitution model GTR, gamma distribution, and estimation of proportion of invariable sites using GTRGAMMAI (Stamatakis 2014).

The genomes for the genome-based analyses were gathered from GenBank, including the sequence for strain Pla110^T^. Completeness and contamination of the genome was determined using CheckM v1.0.131 (Parks et al. 2015). The average nucleotide identity (ANI) was calculated using OrthoANI (Lee et al. 2016), the average amino acid identity (AAI) was computed with the aai.rb script from the enveomics collection (Rodriguez-R and Konstantinidis 2016) and the percentage of conserved proteins (POCP) was determined as previously described (Qin et al. 2014). The *rpoB* nucleotide sequences (encoding the β-subunit of the DNA-dependent RNA polymerase) were taken from the genome annotations and the sequence identities were determined as described before (Bondoso et al. 2013). Upon extracting only those parts of the sequences that would have been sequenced with the described primer set, the alignment and matrix calculation was done with Clustal Omega (Sievers et al. 2011). The genus thresholds for *rpoB* were taken from a previous study (Kallscheuer et al. 2019d).

For the multi-locus sequence analysis (MLSA), the unique single-copy core genome of all analysed genomes was determined with proteinortho5 (Lechner et al. 2011) with the ‘selfblast’ option enabled. The protein sequences of the resulting orthologous groups were aligned using MUSCLE v.3.8.31 (Edgar 2004). After clipping, partially aligned *C*- and *N*-terminal regions and poorly aligned internal regions were filtered using Gblocks (Castresana 2000). The final alignment was concatenated and clustered using the maximum likelihood method implemented by RaxML (Stamatakis 2014) with the ‘rapid bootstrap’ method and 500 bootstrap replicates. The outgroup consisted of concatenated gene sets of strains from the order *Pirellulales*.

## Results and discussion

### Morphological and physiological analyses

**Fig. 1:**
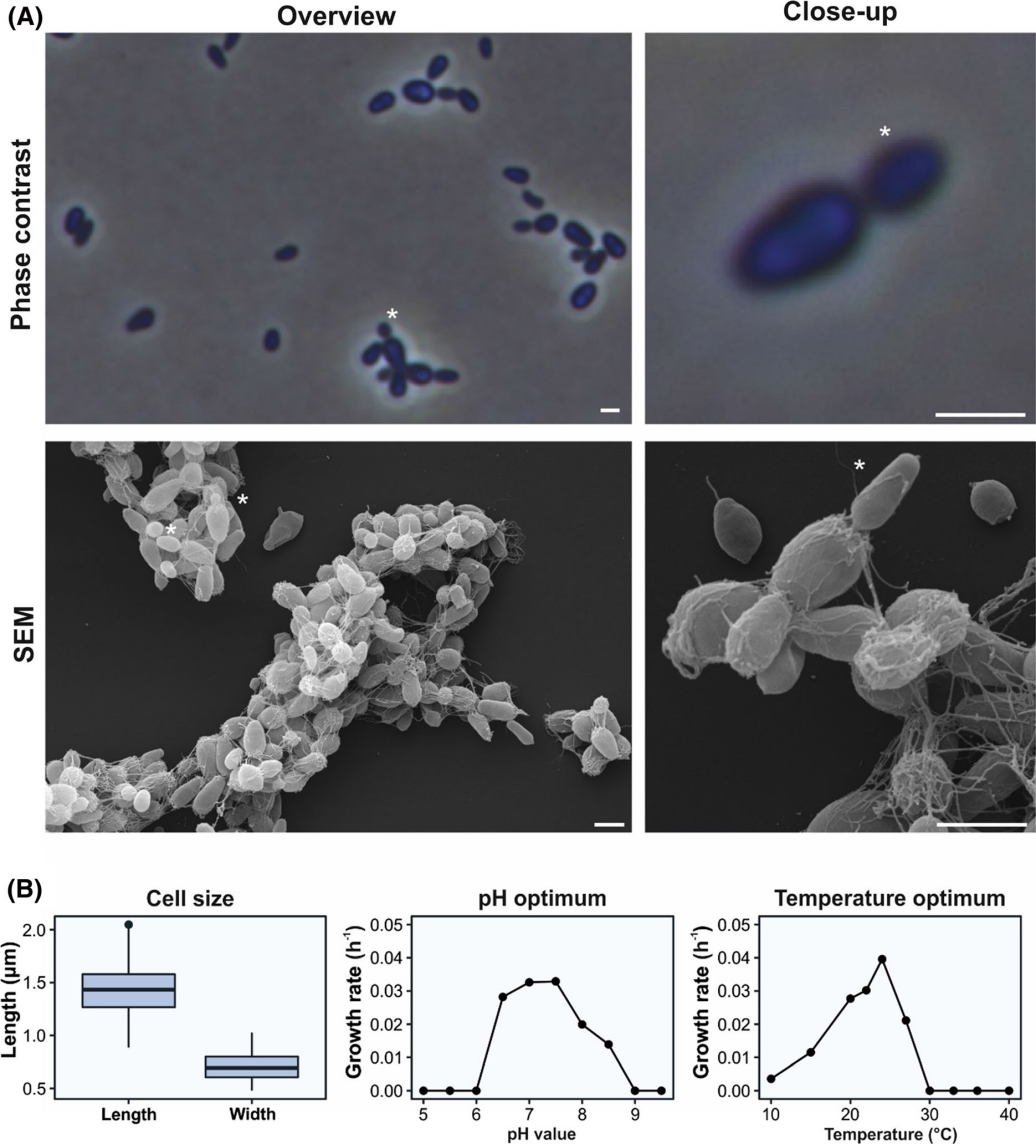
Morphological and physiological characterisation of strain Pla110^T.^ **a** SEM and phase contrast microscopy images showing elongated cells that divide by polar budding with the division site always at the wider side of the cell. Asterisks (*) indicate the nascent bud of a dividing cell. Scale bars represent 1 µm. **b** Boxplot of cell size, and growth rate of strain Pla110^T^ at various pH values and temperatures

Strain Pla110^T^ forms white colonies, while colony colours of related strains range from white to ochre (Table 1). Mature cells of strain Pla110^T^ are attached to each other by loose polar fimbriae, enabling the cells to grow in aggregates or rosettes (Fig. 1a). Fibres originate from crateriform structures, which were also observed on one of the two poles of Pla110^T^ cells.

**Table 1:** Phenotypic and genomic comparison between strain Pla110^T^ and its close relatives *Alienimonas californiensis* CA12^T^ (A), *Fuerstiella marisgermanici* NH11^T^ (B), *Gimesia maris* DSM 8797^T^ (C), *Thalassoglobus neptunia* KOR42^T^ (D), *Planctomicrobium piriforme* DSM 26348^T^ (E), *Planctopirus limnophila* Mu290^T^ (F), *Rubinisphaera brasiliensis* DSM 5305^T^ (G) and *Schlesneria paludicola* MPL7^T^ (H), n.o. not observed

Characteristics	Pla110^T^	A	B	C	D	E	F	G	H
*Phenotypic features*									
Shape	Pear-shaped	Round to pear-shaped	Pear-shaped to ovoid	Ovoid	Round	Ellipsoidal to pear-shaped	Spherical to ovoid	Spherical to ovoid	Ovoid
Cell size (µm)	1.4 ± 0.2 × 0.7 ± 0.1	2.0 × 1.5	1.2–2.5 × 0.9–1.7	1.8 × 0.7	1.6	1.7–2.8 × 0.9–1.3	0.6-1.0 × 0.9–1.6	0.7–1.8	1.7 × 1.1
Colony colour	White	Pink	Cream	Pink	White	Colourless	Pink	Yellow to ochre	Colourless
Aggregates	Yes	Yes	Yes	Yes	Yes	Yes	Yes	Yes	Yes
Division	Polar budding	Budding	Budding	Budding	Budding	Budding	Budding	Budding	Budding
Flagellum	Yes	Yes	Yes	Yes	n.o.	Yes	Yes	Yes	Yes
Crateriform structures	Yes	Polar	Polar	All over	Polar	All over	Yes	Yes	Polar
Fimbriae	Fiber	Polar matrix or fiber	Polar fiber	Fiber	Few fiber	Many	Fiber	Yes	Yes
Capsule	n. o.	n.o.	n.o.	n.o.	n.o.	n.o.	n.o.	n.o.	Yes
Bud shape	Like mother cell	Like mother cell	Like mother cell	Like mother cell	Round	Like mother cell	Like mother cell	Lke mother cell	Like mother cell
Budding pole	Polar	Polar	Polar	Polar	Polar	Polar	Polar	Polar	Polar
Stalk	Yes	n.o.	n.o.	Yes	n.o.	Yes	Yes	Yes	Rare
Holdfast structure	n. o.	n.o.	n.o.	n.o.	n.o.	n.o.	Yes	n.o.	n.o.
*Genomic features*									
Genome size (bp)	6,125,480	5,475,215	8,920,478	7,816,689	6,734,412	6,317,004	5,460,085	6,006,602	8,702,386
G + C content [%]	50.3	70.7	55.9	50.4	52.8	58.8	53.7	56.4	55.7
Coding density	84.3	88.5	87.6	86.9	85.7	85.8	84.9	86.2	84
Completeness	96.55	94.83	95.69	98.28	96.55	95.69	96.55	94.83	96.55
Contamination	3.45	0	1.72	1.72	0	1.72	1.72	3.45	3.45
Transposable elements	5	1	2	4	5	1	3	3	2
Transposable elements /Mb	0.82	0.18	0.22	0.51	0.74	0.16	0.55	0.5	0.23
Total genes	4669	4382	6732	6062	5584	5117	4361	4887	6963
Genes/Mb	762	800	755	776	829	810	799	814	800
Giant genes	1	0	8	8	0	1	1	0	0
Putative proteins	4607	4309	6645	5986	5508	5050	4274	4824	6867
Putative proteins /Mb	752	787	745	766	818	799	783	803	789
Hypothetical proteins	1854	1798	3890	2400	2516	2814	2316	2581	4046
tRNAs	47	65	60	66	70	53	75	50	77
tRNAs/Mb	7.8	11.9	6.7	8.4	10.4	8.4	13.7	8.3	8.9
16S rRNA genes	2	2	2	2	1	1	2	2	1

When examined with phase contrast microscopy, the cells appeared to be pear-shaped and on average 1.4 ± 0.2 by 0.7 ± 0.1 µm in size (Fig. 1b), which is relatively elongated for a planctomycetal cell (Table 1). This characteristic makes them distinguishable from their close relatives. Typical for members of *Planctomycetaceae*, the cells perform cell division by polar budding. The wider side of the cell was always observed to be the origin of the nascent bud during both SEM and phase contrast microscopy (Fig. 1a).

Strain Pla110^T^ was isolated from the surface of plastic (polystyrene), whereas many of its close relatives were found on plant surfaces and can subsist on sugars. The strain was found to grow chemoorganotrophically, to be strictly aerobic and to grow at temperatures ranging from 10 to 27 °C, with the optimum at 24 °C (Fig. 1b). The pH values that permit growth of the strain range from 6.5 to 8.5, with the optimum at 7.5 (Fig. 1b). The growth rate of this strain was calculated to be 0.033 h^− 1^, which corresponds to a doubling time of 21 h.

### Genomic characteristics

Compared to other Planctomycetes, the genome of the novel strain features an average number of transposable elements per Mb (0.82). Proteins per Mb (752), genes per Mb (762) and tRNAs per Mb (7.67) are all at the lower end of the planctomycete spectrum (Table 1). The genome is 6,125,480 bp in length, features two 16S rRNA genes and has a coding density of 84.5%. The G + C content of strain Pla110^T^ is 55.3%, which is in the lower range compared to other members of the family *Planctomycetaceae* (50.4–70.7%, Table 1). The number of transposable elements per Mb (0.82) is higher than for most of its close relatives.

### Phylogenetic analysis

**Fig. 2:**
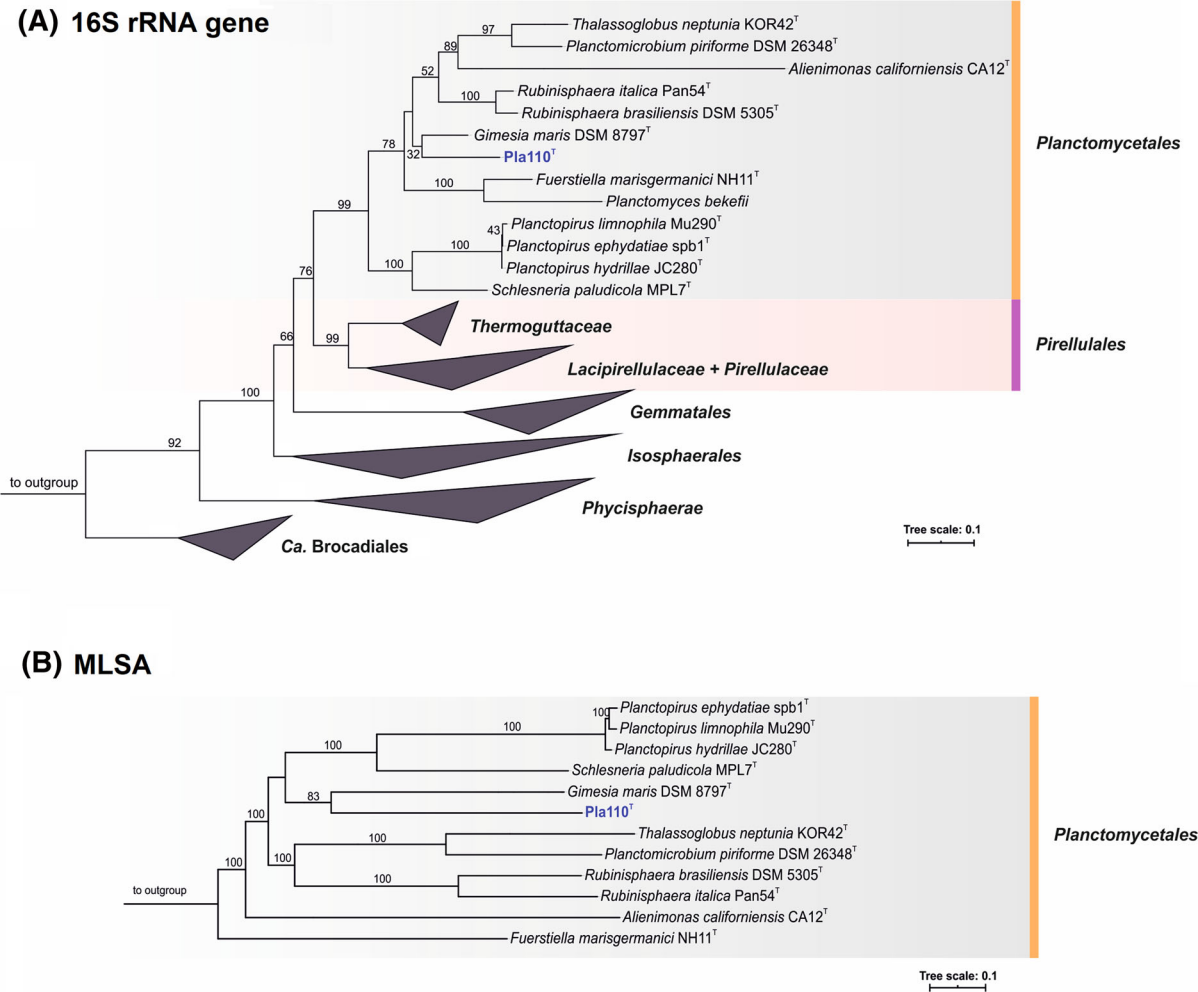
Phylogenetic inference of strain Pla110^T^ **a** 16S rRNA gene-based phylogenetic tree of described Planctomycetes and strain Pla110^T^ indicated in blue. Bootstrap values are indicated as a proportion of 1,000 re-samplings (in %). The outgroup consists of three 16S rRNA genes from the PVC superphylum. **b** Whole genome-based MLSA phylogeny, with bootstrap values based on 500 re-samplings at the nodes (in %). The outgroup consists of several representatives of the order *Pirellulales*. *Planctomyces bekefii* (Dedysh et al. 2020) was included in the 16S rRNA gene-based comparison, but not in the MLSA comparison as only metagenome-assembled genomes are available

**Fig. 3:**
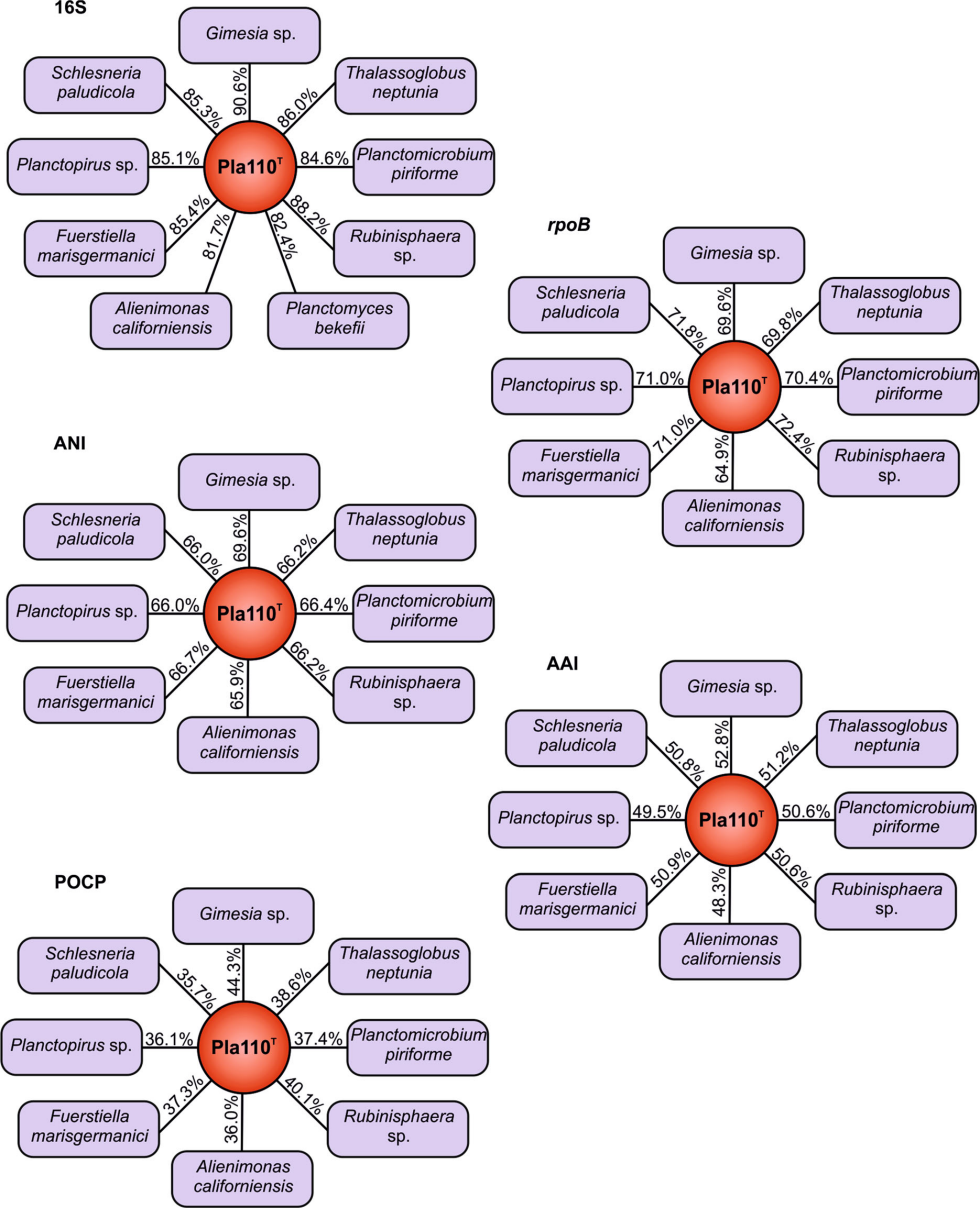
Comparison of phylogenetic markers to separate species and genera To determine strain Pla110^T^ as a novel genus and species, the strain was compared to several related strains. Methods used: 16S rRNA gene sequence identity (16S), average amino acid identity (AAI), *rpoB* gene identity, average nucleotide identity (ANI), and percentage of conserved proteins (POCP). *Planctomyces bekefii* (Dedysh et al. 2020) was included in the 16S rRNA gene comparisons, but not in the others as only metagenomic bins are available

Based on 16S rRNA gene comparison and MLSA, strain Pla110^T^ clusters within the family *Planctomycetaceae* according to the recent re-definition (Dedysh et al. 2019) (Fig. 2). However, it remains elusive from these analyses which genus is the current closest neighbour, although both trees showed a relationship with the type strain of *Gimesia maris*, to which high pairwise 16S rRNA homology was found (90.5%). The 16S rRNA sequence similarities between strain Pla110^T^ and the described genera within the family *Planctomycetaceae* range from 81.7–90.6% (Fig. 3). These identity values are below the suggested threshold for genera of 94.5% (Yarza et al. 2014), indicating that strain Pla110^T^ is not part of any established genus, but instead represents a novel genus.

Other phylogenetic markers, such as *rpoB* similarity (Bondoso et al. 2013), AAI (Konstantinidis and Tiedje 2005) and POCP (Qin et al. 2014) provide additional means to determine if strain Pla110^T^ is indeed part of a new genus (Fig. 3). Comparison of AAI values of strain Pla110^T^ and other members of the family *Planctomycetaceae* result in an identity range between 48.3% and 52.8% (Fig. 3). This range falls below the threshold of 60%, supporting the conclusion that this strain is indeed part of a novel genus (Luo et al. 2014). Comparison of the POCP yielded values of 35.7–44.3% (Fig. 3), which are below the 50% cut-off value, again indicating a separate genus. The similarity of a 1200 bp region of the *rpoB* gene was used previously to infer phylogeny in genera belonging to the family *Planctomycetaceae* (Kallscheuer et al. 2019d). For the novel strain, we observed an identity of the mentioned partial sequence of the *rpoB* gene of 64.9–72.4% to other *Planctomycetaceae* members (Fig. 3). Again, these values are below the proposed threshold range for genera of 75.5–78% (Kallscheuer et al. 2019d), which is in line with the delineation of Pla110^T^ from existing genera.

Taken together, based on results obtained for phylogeny and morphology we conclude that strain Pla110^T^ belongs to a novel genus. We propose the name *Polystyrenella* gen. nov., with *Polystyrenella longa* sp. nov. as type species. Pla110^T^ is the type strain of *Polystyrenella longa*.

#### *Polystyrenella* gen. nov.

*Polystyrenella* (Po.ly.sty.re.nel’la. N.L. neut. n. *polystyrenum* polystyrene; N.L. fem. dim. n. *Polystyrenella* an organism isolated from polystyrene).

The members of this genus have a Gram-negative cell envelope architecture, are aerobic, mesophilic, neutrophilic and heterotrophic. Cells are pear-shaped and divide by polar budding. The genus is part of the family *Planctomycetaceae*, order *Planctomycetales*, class *Planctomycetia*, phylum *Planctomycetes*.

#### *Polystyrenella longa* sp. nov.

*Polystyrenella longa (*lon’ga. L. fem. adj. longa long; corresponding to the longish appearance of the cells).

The members of this species are pear-shaped cells that grow in aggregates or rosettes. The cells are between 1.4 ± 0.2 by 0.7 ± 0.1 µm in size and divide by polar budding. The cells produce fimbriae originating from one of the cell poles, and have crateriform structures on this fibre pole. The type strain grows at 10–27 °C (optimum 24 °C) and pH 6.5–8.5 (optimum 7.5). Forms white colonies on M1H NAG ASW agar. The G + C content of the type strain genome is 50.3%.

The type strain, Pla110^T^ (DSM 103387^T^ = LMG 29693^T^), was isolated from polystyrene particles submerged near Heiligendamm in the Baltic Sea. The genome (accession no. CP036281) and 16S rRNA gene sequence (accession no. MK554533) are available from GenBank.

## References

[CR1] Acehan D, Santarella-Mellwig R, Devos DP (2013). A bacterial tubulovesicular network. J Cell Sci.

[CR2] Bengtsson MM, Øvreås L (2010). Planctomycetes dominate biofilms on surfaces of the kelp *Laminaria hyperborea*. BMC Microbiol.

[CR3] Bengtsson MM, Sjøtun K, Lanzén A, Øvreås L (2012). Bacterial diversity in relation to secondary production and succession on surfaces of the kelp *Laminaria hyperborea*. ISME J.

[CR4] Boedeker C, Schuler M, Reintjes G, Jeske O, van Teeseling MC, Jogler M, Rast P, Borchert D, Devos DP, Kucklick M, Schaffer M, Kolter R, van Niftrik L, Engelmann S, Amann R, Rohde M, Engelhardt H, Jogler C (2017). Determining the bacterial cell biology of Planctomycetes. Nat Commun.

[CR5] Boersma A, Kallscheuer N, Wiegand S, Rast R, Peeters S, Mesman R, Heuer A, Boedeker C, Jetten M, Rohde M, Jogler M, Jogler C (2019) *Alienimonas californiensis* gen. nov. sp. nov., a novel Planctomycete isolated from the kelp forest in Monterey Bay. Antonie van Leeuwenhoek. 10.1007/s10482-019-01367-4 10.1007/s10482-019-01367-4 31802338

[CR6] Bondoso J, Harder J, Lage OM (2013). *rpoB* gene as a novel molecular marker to infer phylogeny in Planctomycetales. Antonie Van Leeuwenhoek.

[CR7] Bondoso J, Balague V, Gasol JM, Lage OM (2014). Community composition of the Planctomycetes associated with different macroalgae. FEMS Microbiol Ecol.

[CR8] Bondoso J, Albuquerque L, Nobre MF, Lobo-da-Cunha A, da Costa MS, Lage OM (2015). *Roseimaritima ulvae* gen. nov., sp. nov. and *Rubripirellula obstinata* gen. nov., sp. nov. two novel planctomycetes isolated from the epiphytic community of macroalgae. Syst Appl Microbiol.

[CR9] Bondoso J, Godoy-Vitorino F, Balague V, Gasol JM, Harder J, Lage OM (2017). Epiphytic Planctomycetes communities associated with three main groups of macroalgae. FEMS Microbiol Ecol.

[CR10] Castresana J (2000). Selection of conserved blocks from multiple alignments for their use in phylogenetic analysis. Mol Biol Evol.

[CR11] Dedysh SN, Kulichevskaya IS, Beletsky AV, Ivanova AA, Rijpstra WIC, Damsté JSS, Mardanov AV, Ravin NV (2019) *Lacipirellula parvula* gen. nov., sp. nov., representing a lineage of planctomycetes widespread in low-oxygen habitats, description of the family *Lacipirellulaceae* fam. nov. and proposal of the orders *Pirellulales* ord. nov., *Gemmatales* ord. nov. and *Isosphaerales* ord. nov. Syst Appl Microbiol 43: 126050 10.1016/j.syapm.2019.126050 PMC6995999 31882205

[CR12] Dedysh SN, Henke P, Ivanova AA, Kulichevskaya IS, Philippov DA, Meier-Kolthoff JP, Goker M, Huang S, Overmann J (2020). 100-year-old enigma solved: identification, genomic characterization and biogeography of the yet uncultured *Planctomyces bekefii*. Environ Microbiol.

[CR13] Devos DP, Reynaud EG (2010). Evolution. Intermediate steps. Science.

[CR14] Devos DP, Jogler C, Fuerst JA (2013). The 1st EMBO workshop on PVC bacteria-*Planctomycetes-Verrucomicrobia-Chlamydiae* superphylum: exceptions to the bacterial definition?. Antonie Van Leeuwenhoek.

[CR15] Devos DP (2014). PVC bacteria: variation of, but not exception to, the Gram-negative cell plan. Trends Microbiol.

[CR16] Edgar RC (2004). MUSCLE: multiple sequence alignment with high accuracy and high throughput. Nucleic Acids Res.

[CR17] Frank O, Michael V, Pauker O, Boedeker C, Jogler C, Rohde M, Petersen J (2014). Plasmid curing and the loss of grip - The 65-kb replicon of *Phaeobacter inhibens* DSM 17395 is required for biofilm formation, motility and the colonization of marine algae. Syst Appl Microbiol.

[CR18] Fuerst JA, Sagulenko E (2011). Beyond the bacterium: Planctomycetes challenge our concepts of microbial structure and function. Nat Rev Microbiol.

[CR19] Fuerst JA, Webb RI (1991). Membrane-bounded nucleoid in the eubacterium *Gemmata obscuriglobus*. Proc Natl Acad Sci USA.

[CR20] Graça AP, Calisto R, Lage OM (2016). Planctomycetes as Novel Source of Bioactive Molecules. Front Microbiol.

[CR21] Jeske O, Jogler M, Petersen J, Sikorski J, Jogler C (2013). From genome mining to phenotypic microarrays: Planctomycetes as source for novel bioactive molecules. Antonie Van Leeuwenhoek.

[CR22] Jeske O, Schüler M, Schumann P, Schneider A, Boedeker C, Jogler M, Bollschweiler D, Rohde M, Mayer C, Engelhardt H, Spring S, Jogler C (2015). Planctomycetes do possess a peptidoglycan cell wall. Nat Commun.

[CR23] Jeske O, Surup F, Ketteniß M, Rast P, Förster B, Jogler M, Wink J, Jogler C (2016). Developing techniques for the utilization of Planctomycetes as producers of bioactive molecules. Front Microbiol.

[CR24] Jogler C, Glöckner FO, Kolter R (2011). Characterization of *Planctomyces limnophilus* and development of genetic tools for its manipulation establish it as a model species for the phylum Planctomycetes. Appl Environ Microbiol.

[CR25] Jogler C, Waldmann J, Huang X, Jogler M, Glöckner FO, Mascher T, Kolter R (2012). Identification of proteins likely to be involved in morphogenesis, cell division, and signal transduction in Planctomycetes by comparative genomics. J Bacteriol.

[CR26] Jogler M, Jogler C, Fuerst JA (2013). Towards the development of genetic tools for Planctomycetes. Planctomycetes: cell structure, origins and biology.

[CR27] Jogler C (2014). The bacterial ‘mitochondrium’. Mol Microbiol.

[CR28] Kallscheuer N, Jogler M, Wiegand S, Peeters SH, Heuer A, Boedeker C, Jetten MS, Rohde M, Jogler C (2019a) *Rubinisphaera italica* sp. nov. isolated from a hydrothermal area in the Tyrrhenian Sea close to the volcanic island Panarea. Antonie van Leeuwenhoek, 10.1007/s10482-019-01329-w 10.1007/s10482-019-01329-w PMC7717053 31773447

[CR29] Kallscheuer N, Jogler M, Wiegand S, Peeters SH, Heuer A, Boedeker C, Jetten MS, Rohde M, Jogler C (2019). Three novel *Rubripirellula* species isolated from plastic particles submerged in the Baltic Sea and the estuary of the river Warnow in northern Germany. Antonie Van Leeuwenhoek.

[CR30] Kallscheuer N, Wiegand S, Jogler M, Boedeker C, Peeters SH, Rast P, Heuer A, Jetten MSM, Rohde M, Jogler C (2019c) *Rhodopirellula heiligendammensis* sp. nov., *Rhodopirellula pilleata* sp. nov., and *Rhodopirellula solitaria* sp. nov. isolated from natural or artificial marine surfaces in Northern Germany and California, USA, and emended description of the genus Rhodopirellula. Antonie van Leeuwenhoek, 10.1007/s10482-019-01366-5 10.1007/s10482-019-01366-5 31802336

[CR31] Kallscheuer N, Wiegand S, Peeters SH, Jogler M, Boedeker C, Heuer A, Rast P, Jetten MSM, Rohde M, Jogler C (2019d) Description of three bacterial strains belonging to the new genus *Novipirellula* gen. nov., reclassificiation of *Rhodopirellula rosea* and *Rhodopirellula caenicola* and readjustment of the genus threshold of the phylogenetic marker *rpoB* for *Planctomycetaceae*. Antonie van Leeuwenhoek, 10.1007/s10482-019-01374-5 10.1007/s10482-019-01374-5 31853689

[CR32] Kallscheuer N, Wiegand S, Heuer A, Rensink S, Boersma AS, Jogler M, Boedeker C, Peeters SH, Rast P, Jetten MS, Rohde M, Jogler C (2020) *Blastopirellula retiformator* sp. nov. isolated from the shallow-sea hydrothermal vent system close to Panarea Island. Antonie van Leeuwenhoek. 10.1007/s10482-019-01377-2 10.1007/s10482-019-01377-2 31894497

[CR33] Kohn T, Heuer A, Jogler M, Vollmers J, Boedeker C, Bunk B, Rast P, Borchert D, Glöckner I, Freese HM, Klenk HP, Overmann J, Kaster AK, Wiegand S, Rohde M, Jogler C (2016) *Fuerstia marisgermanicae* gen. nov., sp. nov., an unusual member of the phylum Planctomycetes from the German Wadden Sea. Front Microbiol 7: 2079 10.3389/fmicb.2016.02079 PMC5177795 28066393

[CR34] Kohn T, Wiegand S, Boedeker C, Rast P, Heuer A, Jetten M, Schüler M, Becker S, Rohde C, Müller R-W, Rohde M, Engelhardt H, Jogler M, Jogler C (2019). *Planctopirus ephydatiae*, a novel Planctomycete isolated from a freshwater sponge. Syst Appl Microbiol.

[CR35] König E, Schlesner H, Hirsch P (1984). Cell wall studies on budding bacteria of the *Planctomyces/Pasteuria* group and on a *Prosthecomicrobium* sp. Arch Microbiol.

[CR36] Konstantinidis KT, Tiedje JM (2005). Genomic insights that advance the species definition for prokaryotes. Proc Natl Acad Sci USA.

[CR37] Konstantinidis Rodriguez-R,LM, KT (2016) The enveomics collection: a toolbox for specialized analyses of microbial genomes and metagenomes. PeerJ Preprints 4: e1900v1

[CR38] Kulichevskaya IS, Serkebaeva YM, Kim Y, Rijpstra IC, Sinninghe Damste JS, Liesack W, Dedysh SN (2012). *Telmatocola sphagniphila* gen. nov., sp. nov., a novel dendriform planctomycete from northern wetlands. Front Microbiol.

[CR39] Lachnit T, Fischer M, Kunzel S, Baines JF, Harder T (2013). Compounds associated with algal surfaces mediate epiphytic colonization of the marine macroalga *Fucus vesiculosus*. FEMS Microbiol Ecol.

[CR40] Lage OM, Bondoso J (2014). Planctomycetes and macroalgae, a striking association. Front Microbiol.

[CR41] Lechner M, Findeiss S, Müller L, Marz M, Stadler P, Prohaska S (2011). Proteinortho: Detection of (Co)Orthologs in Large-Scale Analysis. BMC Bioinformatics.

[CR42] Lee I, Ouk Kim Y, Park SC, Chun J (2016). OrthoANI: An improved algorithm and software for calculating average nucleotide identity. Int J Syst Evol Microbiol.

[CR43] Lindsay MR, Webb RI, Fuerst JA (1997). Pirellulosomes: A new type of membrane-bounded cell compartment in planctomycete bacteria of the genus *Pirellula*. Microbiology-UK.

[CR44] Lonhienne TG, Sagulenko E, Webb RI, Lee KC, Franke J, Devos DP, Nouwens A, Carroll BJ, Fuerst JA (2010). Endocytosis-like protein uptake in the bacterium *Gemmata obscuriglobus*. Proc Natl Acad Sci USA.

[CR45] Luo C, Rodriguez RL, Konstantinidis KT (2014). MyTaxa: an advanced taxonomic classifier for genomic and metagenomic sequences. Nucleic Acids Res.

[CR46] Neumann S, Wessels HJ, Rijpstra WI, Sinninghe Damste JS, Kartal B, Jetten MS, van Niftrik L (2014). Isolation and characterization of a prokaryotic cell organelle from the anammox bacterium *Kuenenia stuttgartiensis*. Mol Microbiol.

[CR47] Overmann J, Abt B, Sikorski J (2017). Present and Future of Culturing Bacteria. Annu Rev Microbiol.

[CR48] Parks DH, Imelfort M, Skennerton CT, Hugenholtz P, Tyson GW (2015). CheckM: assessing the quality of microbial genomes recovered from isolates, single cells, and metagenomes. Genome Res.

[CR49] Peeters SH, van Niftrik L (2018). Trending topics and open questions in anaerobic ammonium oxidation. Curr Opin Chem Biol.

[CR50] Peeters SH, Wiegand S, Kallscheuer N, Jogler M, Heuer A, Jetten MSM, Rast P, Boedeker C, Rohde M, Jogler C (2019). Three marine strains constitute the novel genus and species *Crateriforma conspicua* in the phylum Planctomycetes. Antonie Van Leeuwenhoek.

[CR51] Pilhofer M, Rappl K, Eckl C, Bauer AP, Ludwig W, Schleifer KH, Petroni G (2008). Characterization and evolution of cell division and cell wall synthesis genes in the bacterial phyla *Verrucomicrobia, Lentisphaerae, Chlamydiae*, and *Planctomycetes* and phylogenetic comparison with rRNA genes. J Bacteriol.

[CR52] Pruesse E, Peplies J, Glöckner FO (2012). SINA: accurate high-throughput multiple sequence alignment of ribosomal RNA genes. Bioinformatics.

[CR53] Qin QL, Xie BB, Zhang XY, Chen XL, Zhou BC, Zhou J, Oren A, Zhang YZ (2014). A proposed genus boundary for the prokaryotes based on genomic insights. J Bacteriol.

[CR54] Rast P, Glockner I, Boedeker C, Jeske O, Wiegand S, Reinhardt R, Schumann P, Rohde M, Spring S, Glockner FO, Jogler C, Jogler M (2017). Three Novel Species with Peptidoglycan Cell Walls form the New Genus *Lacunisphaera* gen. nov. in the Family *Opitutaceae* of the Verrucomicrobial Subdivision 4. Front Microbiol.

[CR55] Rensink S, Wiegand S, Kallscheuer N, Rast P, Peeters SH, Heuer A, Boedeker C, Jetten MS, Rohde M, Jogler M, Jogler C (2020) Description of the novel planctomycetal genus *Bremerella*, containing *Bremerella volcania* sp. nov., isolated from an active volcanic site, and reclassification of *Blastopirellula cremea* as *Bremerella cremea* comb. nov. Antonie van Leeuwenhoek, 10.1007/s10482-019-01378-1 10.1007/s10482-019-01378-1 31894496

[CR56] Reva O, Tümmler B (2008). Think big–giant genes in bacteria. Environ Microbiol.

[CR57] Rivas-Marin E, Canosa I, Santero E, Devos DP (2016). Development of Genetic Tools for the Manipulation of the Planctomycetes. Front Microbiol.

[CR58] Rueden CT, Schindelin J, Hiner MC, DeZonia BE, Walter AE, Arena ET, Eliceiri KW (2017). ImageJ2: ImageJ for the next generation of scientific image data. BMC Bioinformatics.

[CR59] Santarella-Mellwig R, Pruggnaller S, Roos N, Mattaj IW, Devos DP (2013). Three-dimensional reconstruction of bacteria with a complex endomembrane system. PLoS Biol.

[CR60] Sievers F, Wilm A, Dineen D, Gibson TJ, Karplus K, Li W, Lopez R, McWilliam H, Remmert M, Söding J (2011). Fast, scalable generation of high-quality protein multiple sequence alignments using Clustal Omega. Mol Syst Biol.

[CR61] Slobodkina GB, Kovaleva OL, Miroshnichenko ML, Slobodkin AI, Kolganova TV, Novikov AA, van Heerden E, Bonch-Osmolovskaya EA (2015). *Thermogutta terrifontis* gen. nov., sp. nov. and *Thermogutta hypogea* sp. nov., thermophilic anaerobic representatives of the phylum Planctomycetes. Int J Syst Evol Microbiol.

[CR62] Stamatakis A (2014). RAxML version 8: a tool for phylogenetic analysis and post-analysis of large phylogenies. Bioinformatics.

[CR63] Strous M, Fuerst JA, Kramer EH, Logemann S, Muyzer G, van de Pas-Schoonen KT, Webb R, Kuenen JG, Jetten MS (1999). Missing lithotroph identified as new planctomycete. Nature.

[CR64] van Teeseling MC, Mesman RJ, Kuru E, Espaillat A, Cava F, Brun YV, VanNieuwenhze MS, Kartal B, van Niftrik L (2015). Anammox Planctomycetes have a peptidoglycan cell wall. Nat Commun.

[CR65] Vollmers J, Frentrup M, Rast P, Jogler C, Kaster AK (2017). Untangling Genomes of Novel Planctomycetal and Verrucomicrobial Species from Monterey Bay Kelp Forest Metagenomes by Refined Binning. Front Microbiol.

[CR66] Wagner M, Horn M (2006). The *Planctomycetes*, *Verrucomicrobia*, *Chlamydiae* and sister phyla comprise a superphylum with biotechnological and medical relevance. Curr Opin Biotechnol.

[CR67] Wiegand S, Jogler M, Jogler C (2018). On the maverick Planctomycetes. FEMS Microbiol Rev.

[CR68] Wiegand S, Jogler M, Boedeker C, Pinto D, Vollmers J, Rivas-Marín E, Kohn T, Peeters SH, Heuer A, Rast P, Oberbeckmann S, Bunk B, Jeske O, Meyerdierks A, Storesund JE, Kallscheuer N, Lücker S, Lage OM, Pohl T, Merkel BJ, Hornburger P, Müller R-W, Brümmer F, Labrenz M, Spormann AM, Op den Camp HJM, Overmann J, Amann R, Jetten MSM, Mascher T, Medema MH, Devos DP, Kaster A-K, Øvreås, L, Rohde, M, Galperin, MY, Jogler, C, 2019. Cultivation and functional characterization of 79 planctomycetes uncovers their unique biology. Nature Microbiology.Wiegand, Jogler S, M, Boedeker, C, Pinto, D, Vollmers, J, Rivas-Marín, E, Kohn, T, Peeters, SH, Heuer, A, Rast, P (2020) Cultivation and functional characterization of 79 Planctomycetes uncovers their unique biology. Nat Microbiology 5: 126–140 10.1038/s41564-019-0588-1 PMC7286433 31740763

[CR69] Yarza P, Yilmaz P, Pruesse E, Glöckner FO, Ludwig W, Schleifer K-H, Whitman WB, Euzéby J, Amann R, Rosselló-Móra R (2014). Uniting the classification of cultured and uncultured bacteria and archaea using 16S rRNA gene sequences. Nat Rev Microbiol.

